# The effect of temporal information among events on Bayesian causal inference in rats

**DOI:** 10.3389/fpsyg.2014.01142

**Published:** 2014-10-08

**Authors:** Kosuke Sawa, Akira Kurihara

**Affiliations:** Department of Psychology, Senshu UniversityKawasaki, Japan

**Keywords:** associative learning, temporal coding, causal inference, causal Bayesian network, rats

## Abstract

A temporal relationship between events of potential cause and effect is critical to generate a causal relationship because the cause has to be followed by the effect. The present study investigated the role of temporal relationships between events in causal inference in rats via Pavlovian pairings. In Experiment 1A, subjects in Group Successive received training trials whereby Event 1 (tone or light) was followed by Events 2 (light or tone) and 3 (sucrose solution), whereas those in Group Simultaneous received simultaneous pairings of Events 1 and 2, and Events 1 and 3. During testing, a lever was inserted into the experimental chamber, where subjects were allowed to press the lever which produced the occurrence of Event 2 without reward. By measuring nose-poke responses during the presentation of Event 2, assumingly based on the prediction of occurrence of sucrose solution, subjects in Group Successive showed a relatively lower response rate than did those in Group Simultaneous. In Experiment 1B, this difference was not observed if subjects received the presentations of Event 2 which was irrelevant to their lever pressing during testing. These results suggest that rats can differentiate their response based on the elemental temporal information even when the integrated temporal map was the same, and implied that rats use temporal information as well as conditional probability based on causal Bayesian network account.

## INTRODUCTION

The issue of causality has been an important and interesting one in philosophy and psychology. The ability to infer causality, in particular, enables organisms to survive in the natural environment by allowing them, for instance, to not only predict the future based on the current status of their surroundings but also to select appropriate behaviors in order to change the environment for specific purposes. In Pavlovian conditioning, whereby subjects receive pairings of conditioned stimuli (CS) and unconditioned stimuli (US) to evoke conditioned responses (CR), organisms are also permitted to predict a future event based on associative knowledge. Despite this superficial similarity between associative and causal knowledge, fundamental differences arise between the two in specific situations.

One of the occasions in which this occurs is when external manipulation intervenes in the causal structure. For example, although we cannot directly observe changes in atmospheric pressure, we can observe changes in scale as measured by a barometer and are able to then predict climate change based on this information. This kind of inference may be derived from associative knowledge between events, but if you artificially manipulated/performed some trick on the barometer to produce a change, you will not be able to predict climate change. Simple associative accounts cannot discriminate between observations of actual barometric change and observations of change based on artificial intervention. A Bayesian network or “graph surgery” would allow us to differentiate between the two, as a causal relationship between atmospheric pressure and barometric change would be removed via an artificial intervention/manipulation performed upon the barometer. Waldmann and Hagmayer (2005) experimentally demonstrated this effect on human causal reasoning, and Blaisdell et al. (2006) reported the possibility that even rats can use causal reasoning strategies based on Bayesian network frameworks when the animals themselves intervene in the causal structure (see also Leising et al., 2008). An important experimental condition of their research involved rats receiving two types of Pavlovian pairings of stimuli, whereby a light was followed by either a sucrose solution or a tone. In the test, subjects in Group Intervene showed less nose-poke responses to the presentation of a tone triggered by lever pressing, whereas subjects in Group Observe (in which the timing of the presentation of the tone was yoked with that in Group Intervene and irrelevant to lever pressing) showed no substantial reductions in nose-poke responses.

These results implied that rats could derive causal information between events from pairing episodes traditionally used in associative learning paradigms. However, numerous studies have shown that rats may acquire associative knowledge based on the Pavlovian pairing of events. Thus, the argument as to whether rats acquire associative or causal knowledge, or how they employ different strategies, associative or causal, to respond in each situation, still seems to be open to debate. One key difference between associative and causal knowledge is that of temporal directionality, in which the cause has to be followed by the effect. Temporal information among events has to be encoded for causal inference, especially for Bayesian network ensembles, as these require directed acyclic graph structures among events.

Temporal coding hypothesis proposed by Matzel et al. (1988) and Barnet et al. (1991, 1997) introduced a possible explanation for how temporal information among events was utilized by animals for generating responses. According to the hypothesis, temporal contiguity is the necessary and sufficient condition for forming associations between events that not only connect events to each other but also encode temporal relationships between them. Knowledge of temporal relationships between events acquired in each trial during training might be integrated into a bigger temporal map, which influences decision-making as to whether subjects should respond. Several researchers have reported how subjects used integrated temporal maps to respond in Pavlovian fear conditioning (e.g., Barnet et al., 1997) and appetitive conditioning (Leising et al., 2007) experiments. A related inference phenomenon was also discovered in spatial learning settings (Blaisdell and Cook, 2005; Sawa et al., 2005).

**FIGURE 1:**
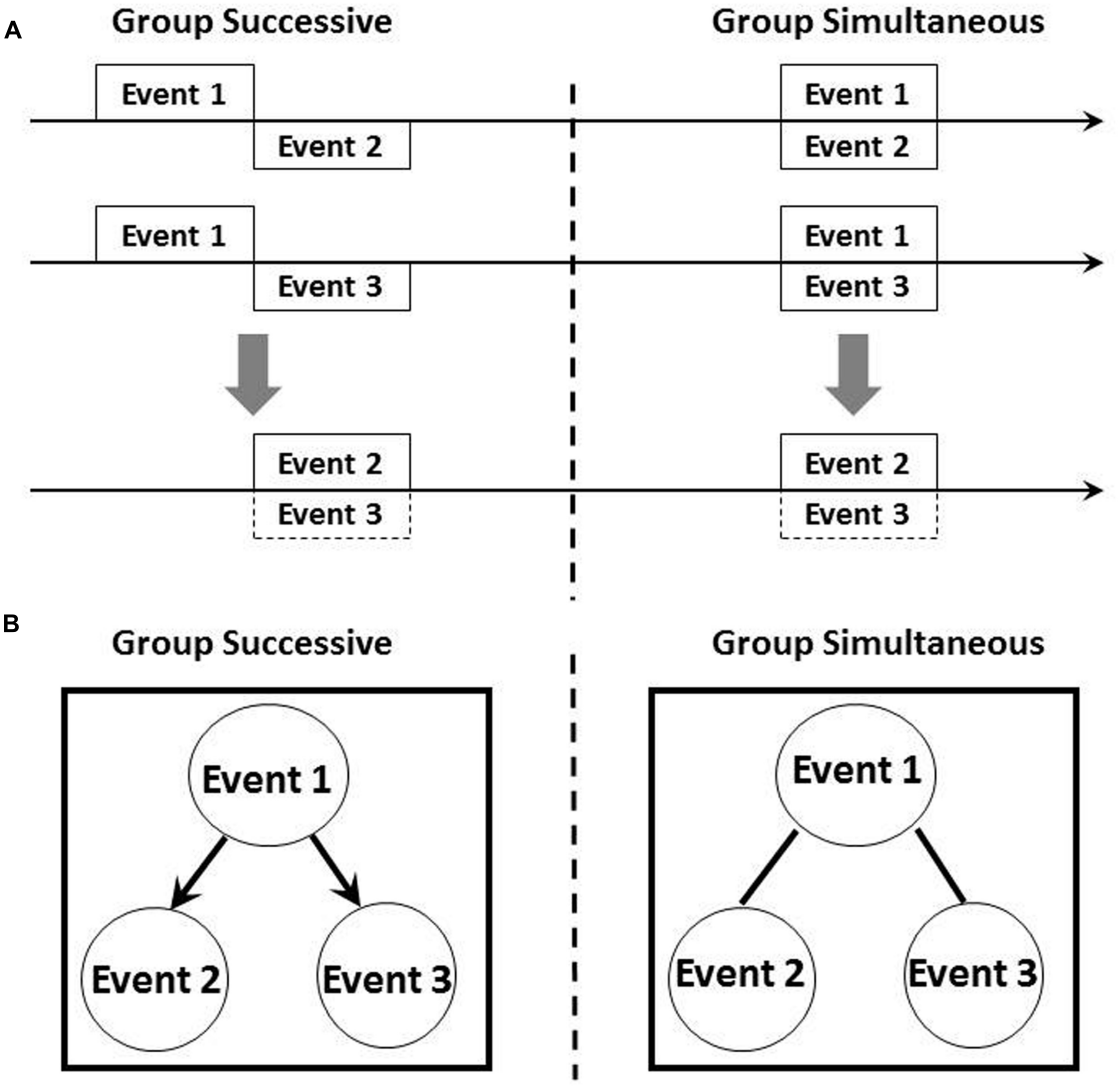
**Schematic representation of temporal relationship **(A)** and hypothetical causal model **(B)** among stimuli in the present experiment.** Horizontal arrow in **(A)** indicated timeline. The upper part of **(A)** illustrated the timing of presentation of each events and the lower part indicated integrated temporal map between Events 2 and 3. Arrows in left side of **(B)** represented causal direction and bars linking events has no causal information.

The left panel of **Figures 1A,B** illustrates the graph structure and temporal diagram of a common causal model examined by present study and also by Blaisdell et al. (2006) in which subjects received successive pairings of Events 1 and 2 and Events 1 and 3 during training. According to temporal coding hypothesis, rats form integrated temporal maps in which Events 2 and 3 occur at the same time. Although inferences based on Bayesian networks allow subjects to predict the occurrence of Event 3 based on their observations of Event 2, an associatively acquired integrated temporal map also permits the prediction of Event 3 based on the occurrence of Event 2. However, if an external intervention produces Event 2, a Bayesian network framework claims that subjects would not be able to predict the occurrence of Event 3 based on the idea of graph surgery, although associative accounts such as those of temporal coding hypothesis enable the prediction of Event 3.

How, then, could subjects generate causal relationships between events and differentiate between the results of associative and causal inference? As we described earlier, a critical feature of causality is the presence of a temporal relationship between cause and effect. Training procedures in which Event 1 precedes Events 2 and 3 are key to generating causality. The present study therefore aims to investigate this point by manipulating temporal properties during training. The right panel of **Figures 1A,B** depicts the graph structure and temporal diagram of a critical procedure we performed in this experiment. During training, subjects received simultaneous pairings of Events 1 and 2, and Events 2 and 3. An integrated temporal map based on temporal coding hypothesis claims that exactly the same results will be observed as for the procedure illustrated in the left panel of **Figures 1A,B**. On the contrary, a Bayesian network approach proposes differing outcomes: subjects cannot generate causality because the temporal rule of cause and effect is broken. First aim of this research was to explore the role of elemental temporal information for causal inference in rats.

In all honesty, the faculty of generating causal relationships in animals, especially in rodents such as rats, needs clarifying. Dwyer et al. (2009) and Dwyer and Burgess (2011) have proposed alternative and parsimonious accounts for these phenomena based on response competition. In response competition accounts, the reason why rats show relatively lower rates of nose-poke responses in critical condition is because lever pressing is incompatible with nose poking (i.e., rats cannot perform lever presses and nose-pokes at the same time). This account is simple yet powerful. The second aim of present study is to explore this possibility. Main claim of response competition account is that rats cannot perform lever pressing and nose-poke at the same time, so that the strength of nose-poke responding of rats in intervening condition might be underestimated relative to those in observing condition. In present study, comparison between two intervening groups in which temporal relationship among events was different, will give some information for this issue.

## EXPERIMENT 1A

In Experiment 1A, we examined the role of temporal relationship among events on causal reasoning. Rats in Group Successive received successive parings of Event 1 and 2 (tone or flashing light), and Event 1 and 3 (sucrose solution) in training as used in Blaisdell et al. (2006), and Group Simultaneous received simultaneous paring of these events, that procedure is not sufficient to generate causality. In testing, subjects in both groups were allowed to press the lever which is immediately followed by Event 2.

### MATERIAL AND METHODS

#### Subjects and apparatus

The subjects were 24 experimentally naïve male Wister rats (Mean body weight: 282.8, SD: 7.09) that were 63 days of age at the beginning of the experimental procedure obtained from CLEA Japan. Throughout the experiment, subjects were housed in plastic cages (42.5 cm in length, 26.5 cm in width, 20 cm in height) with floors covered in paper chips and a stainless-steel grid roof. The cages in which they were housed were kept in the colony room, which was illuminated to a range between 9:00 and 21:00. The room temperature was maintained at 24∘C. Following a week’s acclimation to their home cages, with free access to food and water, all subjects were put on a food deprivation schedule whereby their body weights were maintained at 85% of their free feeding weight.

All experimental procedures were conducted in an operant chamber (30 cm in length, 25 cm in width, 20 cm in height) housed in a sound-and light attenuating isolation box (Med Associates). The walls and ceiling of the experimental chamber were constructed of clear Plexiglas, while its floor consisted of stainless-steel rods. All experimental procedures were conducted with the house light on except for the period of presenting flashing light. The operant chamber was equipped with a dipper for delivering a 20% sucrose solution located at the drinking niche. Speaker for a high-frequency tone (3000 Hz) and a light bulb were also located on the outside walls of the chamber. Levers could be inserted into the cage 4 cm to the left of the food niche and 6 cm above the floor. This research was conducted following the relevant ethics guidelines for research with animals, and was approved by the Institutional Animal Care and Use Committee (IACUC) of Senshu University’s Department of Psychology.

#### Procedures

The experimental design is summarized in **Table 1**. On Day 1, all subjects were acclimated to the operant chamber in a 60-min session, during which they were trained to access and drink the sucrose solution from the dipper. The sucrose solution was delivered according to a discrete uniform distribution (ranging from 5 to 35 s) and the dipper cup was raised for 10 s.

**Table 1:** Experimental design and procedure of Experiment 1A.

Groups	Days 2–5	Days 6–7	Day 8
Simultaneous	Event 1 – Event 2	Event 1 – Event 3	Lever → Event 2
Successive	Event 1 → Event 2	Event 1 → Event 3	Lever → Event 2



*Events 1 and 2: tone or flashing light counterbalanced; Event 3: 20% sucrose.*

Subjects were divided into two equal-sized groups, Group Successive and Group Simultaneous, and received pairing training from Days 2 to 5. Subjects in Group Successive received six trials a day in which Event 1 was followed by Event 2. On the other hand, subjects in Group Simultaneous received six simultaneous pairings of Events 1 and 2 on each day. Events 1 and 2 were the presentation of a tone or flashing light, and the assignment of stimuli was counterbalanced across subjects. Trials occurred with a mean interval of 5 min in daily 60-min sessions. The duration of each event was 10 s.

On Days 6 and 7, first-order conditioning was conducted. In trials involving subjects in Group Successive Event 1 was followed by Event 3 (sucrose solution), whereas those in Group Simultaneous received simultaneous presentations of Events 1 and 3. Daily sessions lasting 60 min were held, in which 12 trials with a 5-min mean inter-trial interval (ITI) were performed.

Levers were inserted into the experimental chambers on Day 8 for testing. All subjects were presented with Event 2 immediately after pressing the lever in 30-min sessions. Nose-poke responses were recorded as an index of subjects’ predictions of sucrose solution.

### RESULTS

Since it showed no lever press responses during testing, one subject in Group Simultaneous was removed from subsequent data analysis except for the analysis on the frequency of lever presses.

**FIGURE 2:**
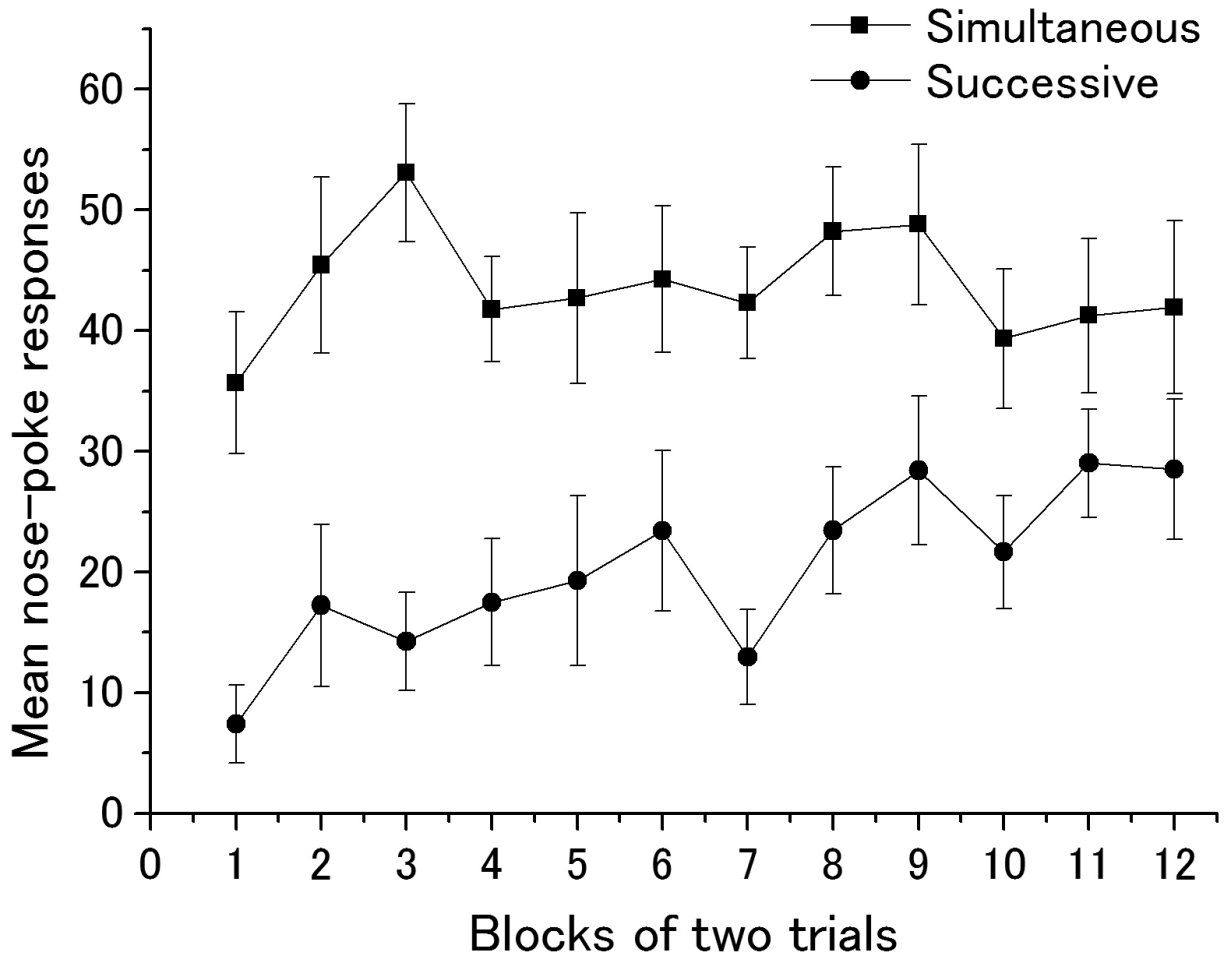
**Mean nose-poke responses of Groups Simultaneous and Successive in acquisition trials.** One block contains two trials. Error bars indicate standard error.

**Figure 2** illustrates the mean nose-poke responses during Event 1 in Group Successive and those during Events 1 and 3 in Group Simultaneous in Phase 2. Visual impressions clearly indicated that Group Simultaneous showed stronger responses than Group Successive although subjects in the latter demonstrated a typical learning curve. A two-factor (groups × blocks) analysis of variance revealed the main effects of groups [*F*(1,22) = 21.60, *p* < 0.01] and the marginal main effect of blocks [*F*(11,242) = 1.82, *p* = 0.052]. Interaction was not significant [*F*(11,242) = 1.21, *p* = 0.28]. Because Event 3 (sucrose solution) was presented with Event 1 in Group Simultaneous, group differences seen in ANOVA were due to the summation effect of unconditioned approach responses to the food niche derived by the sound of a hopper and CR. In order to check whether subjects in Group Successive acquired enough magnitude of nose-poke responding, A Student’s *t*-test was conducted between groups in the last block of Phase 2, which yielded no significant difference, *t*(22) = 1.46.

The mean number of lever presses during testing sessions was 6.83 (SD: 7.00) in Group Simultaneous and 14.08 (SD: 8.24) in Group Successive. A Student’s *t-*test yielded a significant difference between the numbers of lever presses, *t*(22) = 2.22, *p* < 0.05, suggesting that rats in Group Successive pressed the lever significantly more frequently.

**FIGURE 3:**
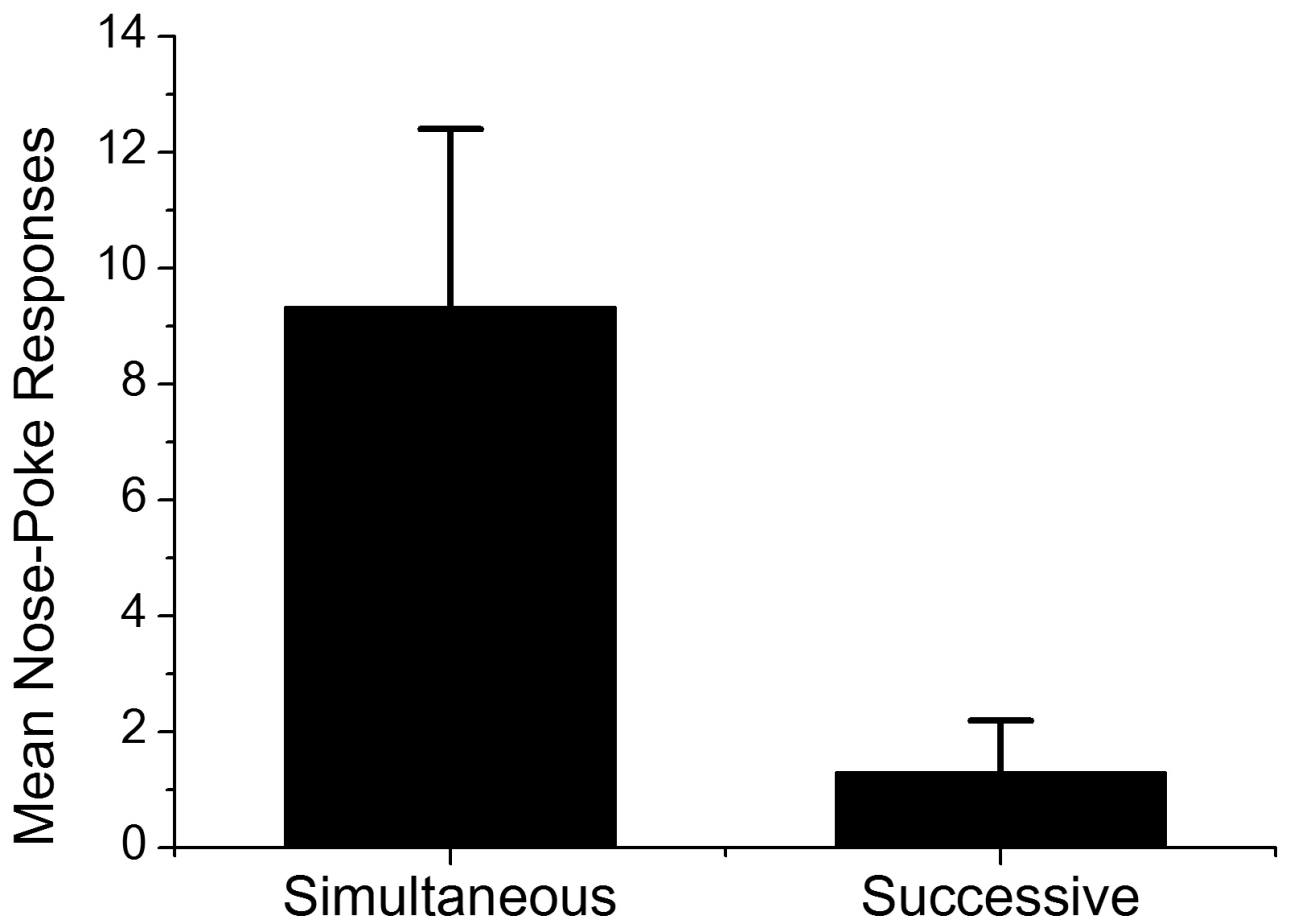
**Mean nose-poke responses of Groups Simultaneous and Successive in first three trials in testing.** Error bars indicate standard error.

**Figure 3** shows the mean frequency of nose-pokes during Event 2 across the first three presentations of stimuli in testing in Groups Simultaneous and Successive. There were two reasons why we adopted the first three trials for analysis. The first was response validity; because non-reinforcing probe trials were administered during testing, conditioned nose-poke responses were extinguished across trials. The second was statistical validity; the numbers of subjects that pressed the lever more than three times (five in the Group Simultaneous and 10 in the Group Successive) were not enough for statistical validation. The results of the Student’s *t*-test on mean nose-poke responses revealed a significant difference between the average nose-poke responses of both groups [*t*(21) = 2.61, *p* < 0.05], which suggested that subjects in Group Simultaneous predicted the occurrence of Event 3, the delivery of sucrose solution, with a higher rate than those in Group Successive. In order to explore the effect of the number of lever presses, correlation analysis between nose-poke responses in first three trials and total numbers of lever presses was conducted. Pearson’s correlation was -0.11 (*p* = 0.75) in Group Simultaneous and 0.51 (*p* = 0.09) in Group Successive. The results of the correlation analysis implied that the number of lever presses had no direct impact on the rate of nose-poke responses.

## EXPERIMENT 1B

Although statistical analysis yielded the difference between Groups Simultaneous and Successive, it is difficult to conclude this difference was due to the situation where voluntary lever pressing was followed by Event 2. Groups Sim-Control and Suc-Control were set for rule out this confounding. Groups Sim-Control and Suc-Control received the same training procedure with Groups Simultaneous and Successive in Experiment 1A, respectively, and then received different procedure in testing with those. In testing, all subjects in Experiment 1B were presented with the lever, but the timings of presentation of Event 2 depended on the timing of lever pressing seen in Experiment 1A (yoked procedure across experiments). If only the training history was crucial and voluntary lever pressing was irrelevant to the observed difference in nose-poke responding in Experiment 1A, subjects in Group Sim-Control should show more nose-pokes than those in Group Suc-Control.

### MATERIAL AND METHODS

#### Subjects and apparatus

Sixteen experimentally naïve male Wister rats were served as subjects obtained from CLEA Japan, that were 63 days of age at the beginning of experiment (Mean body weight: 279.0, SD: 5.01). Situation of housing and apparatus used in experimental procedure were identical with Experiment 1A. This research was conducted following the relevant ethics guidelines for research with animals, and was approved by the IACUC of Senshu University’s Department of Psychology.

#### Method

Subjects were divided into two equal-sized groups, Group Sim-Control and Group Suc-Control. Subjects in Group Sim-Control and Group Suc-Control were received identical procedure from Day 1 to Day 7 with those in Group Simultaneous and Group Successive in Experiment 1A, respectively. In testing, Day 8, subjects in Group Sim-Control and Suc-Control were allowed to press the lever without any consequences. Instead, subjects in those two groups received three trials of presentation of Event 2 with the timing of first three pressing the lever by subjects in Group Simultaneous and Successive in Experiment 1A. Eight subjects each were randomly selected from Group Simultaneous and Successive and the recorded timing of lever pressing in testing session were used as the timing of presentation of Event 2 in Groups Sim-Control and Suc-Control.

### RESULT

Mean frequency of nose-pokes in last two trials in Phase 2 in Group Sim-Control and Suc-Control were 33.3 (SE: 8.76) and 25.2 (SE: 5.78) respectively, which were almost same strength with the result in Experiment 1A. Mean frequency of nose-pokes during Event 2 across the first three presentations of stimuli in testing in Groups Sim-Control and Suc-Control were 2.42 (SE: 0.93) and 2.71 (SE: 1.10). Student’s *t*-test revealed no significant difference between groups [*t*(14) = 0.20]. This result suggested that observing Event 2 was not sufficient to differentiate the nose-poke responding and that voluntary lever press played important role for the difference between Groups Simultaneous and Successive.

## DISCUSSION

The present study aimed to explore the effects of temporal information between events on causal reasoning. We found that a successive relationship could serve as a cue for causality, whereas a simultaneous relationship could not. The results obtained suggested that rats might use temporal relationships between events for reasoning in a different way than previously hypothesized.

According to traditional accounts of associative learning, a bidirectional associative link is formed and activation of representation spreads via associative links to evoke responses based on the properties of a stimulus representation. However, temporal coding hypothesis has introduced a different assumption: subjects will respond based on predictive relationships among events acquired through the integration of elemental temporal maps. If this were the case, the rats in all groups in the present experiment would have shown similar performances, as the temporal relationship between Events 2 and 3 was the same across the groups based on the integrated temporal map. Because procedural differences between Groups Simultaneous and Successive were the temporal relationships between Events 1 and 2 and Events 1 and 3, the role of Event 1 as mediator seems to be crucial in the results of the present study. Although previous research supports the ability of rats to integrate elemental temporal maps in generating predictive responses (e.g., Barnet et al., 1997), the present results suggested different tendency; rats could show the different trend of results based on the elemental temporal information even when the integrated temporal maps were the same.

Another important implication was that elemental temporal information could differentiate rats’ predictive responses to the event following lever presses. According to previous research and Bayesian net accounts (e.g., Blaisdell et al., 2006), even rats might conduct causal reasoning and switch their behaviors if their own responses intervene with the causal model. Although subjects in Group Simultaneous and Successive in the present study were allowed to press the lever, which was immediately followed by Event 2, the rats demonstrated a different trend in the rate of nose-poke responses. In Group Successive, rats received successive pairings, Event 1 → Event 2 and Event 1 → 3, that would enable them to acquire a common causal model. On the other hand, subjects in Group Simultaneous were given simultaneous pairings of events, leading to the construction of another type of model. In the model meant to be formed by Group Simultaneous, every event occurred at the same time, and this temporal relationship did not fit the rule of causality, which specifies that the cause has to be followed by the effect. This logic implies that rats could discriminate whether or not a causal relationship might exist based on the elemental temporal relationship between events. Based on the causal Bayesian network account, suggested in Blaisdell et al. (2006), crucial factor for inference is conditional probability for calculating the Bayes theorem. In present experiment, procedural difference between Groups Simultaneous and Successive was temporal relationship among stimuli, but not the probabilistic relationship. If subjects calculated conditional probability among events based on given trials in training for causal reasoning, temporal property has to have impact on this calculation, according to the present results. Further research would be need for investigating the relationship between temporal information and the perception of probability.

In Experiment 1B, both of Groups Sim-Control and Suc-Control showed the same response tendency of nose-poking. This suggested that the voluntary lever pressing yielding Event 2 played critical role for different responding during testing seen in Experiment 1A. However, one potential problem is that these groups showed relatively weak response in testing. In previous research (e.g., Blaisdell et al., 2006), one important result was the difference between the group in which subjects intervene in the causal model to yield event and the group in which subjects just observe the occurrence of the event. In present experiment, this comparison would be parallel with the comparison between Group Successive and Group Suc-Control. Since these two groups received experimental procedure in different period, direct statistical analysis was not conducted, though Group Successive showed relatively weak responding than Group Suc-Control, which was consisted with the result reported in Blaisdell et al. (2006). One possible reason why only weak responding was observed in Experiment 1B is that only first three trials were obtained, although Blaisdell et al. (2006) used all trials for analyzing. Unlike the situation in Experiment 1A, where subjects voluntary respond to the lever and receive presentations of event, it might not enough to acclimate with settings for subjects in Experiment 1B because “first three trials” occurred very early period of session. Main purpose of Experiment 1B was to rule out the possibility that temporal relationship among events during training could differentiate the test performance which was observed in Experiment 1A. Further support of reliability should be needed even though we could confirm there was no difference between groups.

Another aim of present research was to explore the possibility of response competition account proposed by Dwyer and Burgess (2011) and Dwyer et al. (2009). In present result, subjects in Group Successive and Simultaneous showed different tendency on nose-poking during testing even though all subjects received same procedure in testing. However, more numbers of lever presses were seen in Group Successive than the Simultaneous, which suggested response competition account could be true. On the other hand, as our correlation analysis indicated, there were no significant correlations between numbers of lever presses and nose-pokes; there was also a marginally significant positive correlation in Group Simultaneous, which implied that subjects who pressed the lever more frequently showed stronger nose-poke responding. Although this logic seems to still be indirect evidence to rule out response competition accounts, subjects under the condition whereby lever pressing was followed by a secondary cue for food presentation showed differential rates of predictive behavior based on temporal information. Further investigations to determine the influential conditions or variables of causal cognition are thus necessary.

## AUTHOR CONTRIBUTIONS

Kosuke Sawa developed the design of experiment, analyzed the data and wrote first draft. Akira Kurihara conducted the experiment. Both author contributed equally for later version of paper.

## Conflict of Interest Statement

The authors declare that the research was conducted in the absence of any commercial or financial relationships that could be construed as a potential conflict of interest.
